# Action of melatonin in primary culture of canine mammary tumors

**DOI:** 10.1186/1753-6561-7-S2-P37

**Published:** 2013-04-04

**Authors:** Juliana R Lopes, Bruna V Jardim, Larissa B Maschio, Marina G Moschetta, Thaiz F Borin, Lívia C Ferreira, Naiane N Gonçalves, Camila Leonel, Gabriela B Gelaleti, Debora APC Zuccari

**Affiliations:** 1 Department of Biology, Postgraduate Program in Genetics/Universidade Estadual Paulista - UNESP/IBILCE, São José do Rio Preto (SP) Brazil; 2 Department of Molecular Biology – Laboratory of Molecular Cancer Investigation, Faculdade de Medicina de São José do Rio Preto, FAMERP, São José do Rio Preto (SP) Brazil; 3 Department of Molecular Biology, Postgraduate Program in Health Science/Faculdade de Medicina de São José do Rio Preto, FAMERP, São José do Rio Preto (SP) Brazil

## Background

Breast neoplasms are the most common tumors in female dogs, representing about 50% of all cancers in this animal population. The identification of therapeutic agents that can be used as an alternative treatment for this tumor type has proven to be useful. The administration of melatonin, a hormone secreted by the pineal gland, can exercise oncostatic effects in several types of cancer. Regarding breast cancer its importance is significant. Due to the many similarities shared by humans and dogs, canine mammary tumors are an excellent experimental model. Thus, the aim of this study was to evaluate the effects of melatonin treatment in canine mammary tumors.

## Materials and methods

Ten tumor fragments of female dogs were collected and stored in transport medium for performing the cell cultivation and cell treatment with melatonin. The samples was cultured in medium DMEM (E) and incubated at 37°C in 5% CO_2_. It was established two groups: Control group (untreated) and Group treated with different concentrations of melatonin (0,5mM, 1mM, 2mM, 5mM and 10mM). Cell viability was verified by MTT assay. The results were analyzed evaluating the mean and standard error.

## Results

Melatonin was able to reduce cell viability at all concentrations tested. 60% of samples showed a greater reduction in cell viability when cells were treated with 10mM melatonin.

## Conclusions

Our results suggest that melatonin decreases the viability of the canine mammary neoplasic cells, where, the treatment with 10mM was more effective,treading a promising way to the use as a therapeutic agent in cancer treatment.

## Financial support

FAPESP and CNPq.

